# People’s Ability to Detect Objects Using Click-Based Echolocation: A Direct Comparison between Mouth-Clicks and Clicks Made by a Loudspeaker

**DOI:** 10.1371/journal.pone.0154868

**Published:** 2016-05-02

**Authors:** Lore Thaler, Josefina Castillo-Serrano

**Affiliations:** Department of Psychology, Durham University, Durham, United Kingdom; University of Exeter, UNITED KINGDOM

## Abstract

Echolocation is the ability to use reflected sound to obtain information about the spatial environment. Echolocation is an active process that requires both the production of the emission as well as the sensory processing of the resultant sound. Appreciating the general usefulness of echo-acoustic cues for people, in particular those with vision impairments, various devices have been built that exploit the principle of echolocation to obtain and provide information about the environment. It is common to all these devices that they do not require the person to make a sound. Instead, the device produces the emission autonomously and feeds a resultant sound back to the user. Here we tested if echolocation performance in a simple object detection task was affected by the use of a head-mounted loudspeaker as compared to active clicking. We found that 27 sighted participants new to echolocation did generally better when they used a loudspeaker as compared to mouth-clicks, and that two blind participants with experience in echolocation did equally well with mouth clicks and the speaker. Importantly, performance of sighted participants’ was not statistically different from performance of blind experts when they used the speaker. Based on acoustic click data collected from a subset of our participants, those participants whose mouth clicks were more similar to the speaker clicks, and thus had higher peak frequencies and sound intensity, did better. We conclude that our results are encouraging for the consideration and development of assistive devices that exploit the principle of echolocation.

## Introduction

Echolocation is the ability to use reflected sound to obtain information about the spatial environment. Echolocation has been studied extensively in various bat species, as well as in some marine mammals. It has also been studied in humans. To echolocate a person emits a sound, e.g. a mouth click, and then uses sound reflections to obtain information about the environment. In this way echolocation is an active process that requires both the production of the emission as well as the sensory processing of the resultant sound. People can use echolocation to determine distance, direction, size, material, motion or shape of distal ‘silent’ surfaces (for reviews see [1–3]). In this way it can provide sensory information otherwise unavailable without vision and therefore, direct sensory benefits for people who are blind. For people with vision impairments, the use of echolocation is also associated with benefits in daily life, such as better mobility in unfamiliar places [4]. Going beyond direct sensory benefits, it has also been suggested that the use of echolocation may improve the calibration of spatial representations for people who are blind from an early age [5].

Appreciating the general usefulness of echo-acoustic cues for people, in particular those with vision impairments, various devices have been built that exploit the principle of echolocation to obtain and provide information about the environment [6–15]. Some of these devices are distance measures or localization devices; that is, these devices send out an ultrasonic pulse and then transform the incoming information into a secondary signal about distance and location, which is then fed back to the user. Other devices (e.g., [14]) are based on the idea that the signal should not be changed but that the user’s brain ‘should do the work’. This device sends out an ultrasonic emission, and receives the echoes binaurally via artificial pinnae, and then simply down-samples the signal and sends this down-sampled (but otherwise 'raw') signal to the user via headphones. In this way, it is up to the user to extract the relevant information from the signal. It is common to all these devices that they do not require the person to make a sound. Instead, the device produces the emission autonomously and feeds the resultant sound back to the user.

In the context of auditory processing, people typically show a phenomenon that is referred to as echo-suppression [16], [17]. It refers to a wide class of phenomena according to which, if two sounds are presented in rapid succession, the percept is dominated by the leading sound. As a consequence, the percept of the second sound is suppressed. This can improve speech intelligibility as well as localization of sound sources in conditions in which reverberations are present. Importantly, using a virtual auralization technique it has been suggested that during echolocation where people actively produce the emission making mouth-clicks, echo suppression is reduced as compared to echolocation where people do not actively produce the emission [18]. Importantly, if this result also applied in ‘natural’ conditions, there would be implications for assistive technology. Specifically, since the use of assistive devices based on echolocation does not require people to actively make a sound, there is the chance that people might be at a disadvantage (i.e. their echolocation ability might be reduced) when using a device as compared to making their own emissions. Thus, here we tested if echolocation performance in a simple object detection task was affected by the use of a head-mounted loudspeaker as compared to active clicking. Current devices based on echolocation provide sound to the listener using earphones. In our loudspeaker condition, however, we used only a loudspeaker, but no earphones. We did this to keep the natural hearing experience constant across conditions (i.e. HRTF, frequency response characteristics of the outer and inner ear, real-time listening).

We found that a sample of 27 sighted people new to echolocation did equally well or even better using the loud speaker. We also found that two blind people with expertise in echolocation performed equally well with the speaker and making their own clicks. Finally, we found that even though the two blind experts performed generally better than the sighted participants, the difference in performance was only significant when using mouth clicks. In this way, using the speaker enabled sighted ‘novices’ to approach performance of echo-experts. A correlational analysis of acoustic features of mouth clicks of a subset of our participants (N = 16) showed that clicks that were more similar to the clicks made by the loudspeaker and that therefore had higher intensity and higher peak frequencies were associated with better performance in our experiment.

We discuss the results with respect to previous findings that suggested that echo suppression should be reduced (and echolocation therefore be enhanced) when people make their own clicks. We conclude that our results are encouraging for the consideration and development of assistive devices that exploit the principle of echolocation.

## Method

All procedures were approved by the ethics board in the department of psychology at Durham University and followed the principles laid out by the WHO in the declaration of Helsinki and the BPS code of practice. Blind participants were given accessible versions of all documents. We obtained written informed consent from all participants.

### Overview of the Experiment

Sighted blindfolded and blind participants were asked to use click-based echolocation to determine if there was a disk in front of them or not. The disk could be presented at two different distances (1m and 2m). Participants either echolocated using mouth clicks or using clicks played through a head-worn loudspeaker.

### Participants

For this experiment 27 sighted and 2 blind participants took part. Sighted participants (14 female; mean age: 29.1; SD: 10.1) reported to have normal or corrected to normal vision and hearing and no prior experience with echolocation. Blind participants were both totally blind at time of testing and reported using mouth-click based echolocation on a daily basis. (B1: male, 49 years at time of testing; enucleated in infancy because of retinoblastoma; reported to have used echolocation as long as he can remember. B2: male, 31 years at time of testing; lost sight gradually from birth due to Glaucoma. Since early childhood (approx 3 yrs) only bright light detection; reported to have used echolocation on a daily basis since he was 12 years old). Participants volunteered to take part in the study and were compensated £6/hour or with participant pool credit.

### Apparatus

The experiment was conducted in a sound-insulated and echo-acoustic dampened room (approx. 2.9m x 4.2m x 4.9m, noise-insulated room-inside-a-room construction, lined with acoustic foam wedges that effectively absorb frequencies above 315 Hz).

Participants were seated in the centre of the room on a height-adjustable chair facing the back of the room. In trials where an object was present, participants were presented with a 60cm-diameter disc made of polystyrene covered in aluminium foil mounted on a metal pole (1cm diameter). On trials were an object was absent, participants were presented only with the 1cm diameter metal pole (i.e. the pole from which the disc had been removed). The pole had a movable base to facilitate placing it at either 1m or 2m from the participant. Once participants were seated on the chair, the height was adjusted in order to match the height of participant’s ears with the height of the centre of the disk.

Throughout the experiment participants wore a blindfold and head strap with a loudspeaker mounted on it (Visaton SC5.9 ND; 60g; 90mm (H) x 50mm (W) x 30mm (D)). The speaker was driven by an IBM Lenovo N500 laptop (Intel Pentium Dual PCU T3400 2.16 GHz, 3 GB RAM, 64 bit Windows 7 Enterprise SP1 a), connected via USB Soundcard (Creative Sound Blaster X-Fi HD Sound Card; Creative Technology Ltd., Creative Labs Ireland, Dublin, Ireland) and amplifier (Dayton DTA-1) to the speaker, using Audacity software (Audacity 2.1.0). The speaker was placed on the forehead with its centre placed about 25cm from either ear.

### Sound Characteristics

**Fig 1:**
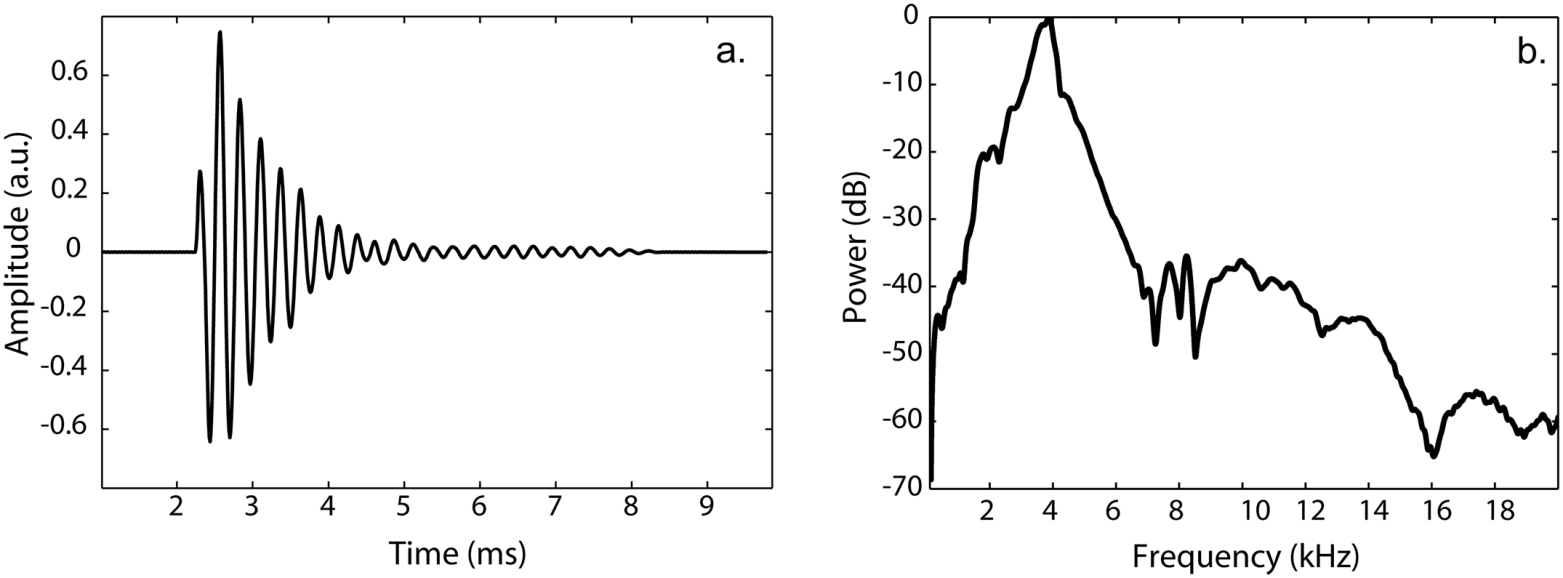
**(a)** Waveform of an individual click as played through the speaker (recorded with DPA SMK-SC4060 with protective grid removed and TASCAM DR100-MKII at 24bit and 96kHz) **(b)** The click’s frequency spectrum.

The sound file (wav-file) used to generate clicks via the speaker had been generated in MatlabR2012 (The Mathworks, Natick, MA) at 24 bit and 96kHz. It was 12.1 seconds long, and contained 17 individual clicks separated by 750 milliseconds of silence. Each individual click was a 4kHz tone amplitude modulated by a decaying exponential. An illustration of the waveform of an individual click as played through the speaker (recorded with DPA SMK-SC4060 (with protective grid removed) and TASCAM DR100-MKII at 24bit and 96kHz) is shown in Fig 1a. The click’s frequency spectrum is shown in Fig 1b. We chose this specific sound for three reasons. First, it has been suggested previously that a sinusoide amplitude modulated by a decaying exponential would be a suitable model for waveforms created by echolocators mouth-clicks [19]. Second, the duration and spectral frequency were within the range of durations and frequencies for echolocation mouth-clicks described previously [20]. Finally, to the experimenters this sound phenomenologically resembled mouth-clicks that people make who echolocate on a regular basis.

The mouth clicks people made varied from person to person, but they all were brief transients. The rate of clicking was comparably across oral and speaker conditions. We recorded clicks for B1 and B2 as well as 14 sighted participants. Unfortunately, we were not able to make recordings for the other sighted participants. Table 1 lists acoustic features of people’s clicks. Clicks were analyzed in Matlab as follows: First, we detected individual clicks by detecting the peak value of the sound envelope computed as absolute value of the waveform. Peaks had to have a minimum separation from one another of 100ms. We then extracted the sound from the peak up to 15ms prior to the peak and 30 ms after. We then fitted exponentials of the form *y* = *ce*^−*bt*^ to the envelope data, where *y* is the fitted envelope data point, and *t* is the sample number. We fitted one curve to the 15ms of envelope data from the beginning to the peak, and one to the 30ms of data from the peak to the end. The fitted curve will be maximal at the peak and drop off as it goes away from the peak. The height of the maximum will depend on *c*, and the drop off rate on *b*. The onset and offset of the sound was defined as the sample where the value of the fitted curve was lower than 95% of the maximum value of the fitted curve. Each click and curve-fit was checked audio-visually and data were rejected if the extracted sound was not a click (e.g. coughing, background noise, swallowing). We then used onset and offset values to extract the click from the sound file and to estimate duration, peak intensity, RMS intensity, and peak frequency (i.e. frequency with maximum amplitude in frequency spectrum) of clicks. We subsequently also computed a ‘dissimilarity measure’ (DM) that quantified how similar the acoustics of a participant’s mouth click was to the speaker click. To compute dissimilarity we first computed the difference between mouth click and speaker click with respect to peak intensity, peak frequency and duration. We did not use RMS intensity because it was highly correlated with peak intensity and because peak intensity by itself had a higher correlation to performance (compare Table 1 and see also ‘Results‘). We then normalized these difference values for each acoustic feature by their standard deviation across participants. We then took the absolute values of these normalized differences. Finally, to get a single dissimilarity measure, we added the normalized absolute difference values together. We did this using only intensity and frequency (DM_I,F_), and using intensity, frequency and duration (DM_I,F,D_).

**Table 1:** Acoustic features of clicks. For reference, features of clicks made by the loud speaker and computed using our methods are given in the top row. Values are means. Standard deviations are given in parenthesis. The last two columns are values of the Dissimilarity Measure (DM) based on differences between mouth clicks and the speaker clicks in terms of peak intensity (I), frequency (F) or duration (D).

Subject	Duration (ms)	RMS Intensity (dB)	Peak Intensity (dB)	Peak Frequency (Hz)	DM_I,F_	DM_I,F,D_
**Speaker**	6.2 (0.1)	-9.9 (0)	-4.4 (0)	3979 (4)	--	--
**B1**	5.3 (1.6)	-10.2 (1.5)	-3.6 (1.4)	3487 (598)	0.8	1
**B2**	4.1 (1.3)	-10.4 (1.6)	-3.6 (1.5)	2903 (378)	1.6	2.1
**S1**	11.6 (4.3)	-21.6 (2.3)	-15.9 (2)	1592 (138)	5.6	6.8
**S2**	11 (5.6)	-24.8 (2.1)	-17.7 (1.5)	2124 (1230)	5.2	6.3
**S3**	5.5 (2.9)	-21.7 (2.7)	-16.3 (2.3)	1834 (503)	5.3	5.5
**S4**	6.2 (4.1)	-21.1 (3.4)	-15 (2.5)	1361 (736)	5.7	5.7
**S5**	4.7 (2.2)	-20.1 (2.4)	-14.7 (2)	2852 (2852)	3.6	4
**S6**	7.2 (2)	-18.6 (2.8)	-13.3 (2.4)	1723 (131)	4.9	5.1
**S7**	6.4 (2.2)	-20.3 (2.9)	-14.7 (2.6)	2094 (272)	4.7	4.7
**S8**	6.6 (2)	-18 (2.3)	-12.6 (2.1)	1472 (179)	5.1	5.2
**S9**	12.8 (1.5)	-8.8 (1.5)	-3.4 (1.6)	1229 (19)	3.9	5.4
**S10**	6 (3.5)	-22.7 (1.9)	-16.6 (1.5)	3149 (316)	3.6	3.7
**S11**	16.1 (6.4)	-24.2 (1.8)	-17.2 (1.5)	1315 (963)	6.2	8.5
**S12**	3.4 (1.4)	-14.8 (2.8)	-9.7 (2.4)	1757 (839)	4.1	4.7
**S13**	18.1 (3.2)	-18.5 (3.1)	-13.2 (3.1)	1015 (40)	5.8	8.5
**S14**	10.8 (3.7)	-18.3 (2.5)	-12.1 (2.2)	1781 (226)	4.6	5.6

### Procedure

For sighted participants the experiment consisted of two sessions. In each session there were two click conditions (self-produced mouth clicks and loud-speaker clicks). The order of click conditions was counterbalanced across participants. In each session, participants completed 48 trials per click condition, with 24 trials for each distance (1m or 2m). The object was absent for 12 out of those 24 trials. The order of distances (1m vs. 2m) and objects (present vs. absent) was block-randomized. In the beginning of each session, the experimenter demonstrated how to make mouth clicks. Participants then practiced until they produced adequate clicks for the task. Our criteria for adequate clicks were (a) that they did not produce ‘double-clicks’ (i.e. clicks that are created when the tongue is quite back in the mouth and basically creates two brief successive oral vacuum pulses, that sound like a deeper ‘clucking’ sound), and (b) that they could make the clicks with comfort and sustain them throughout a 12 second trial at a rate similar to the speaker. Participants completed 2 practice trials per distance and presence condition. They received feedback during practice trials.

For blind participants trained in echolocation the experiment consisted of only one session during which all conditions (speaker vs. mouth clicks; 1m vs. 2m; absent vs. present) were presented in block randomized order.

At the beginning of each trial, participants occluded their ears using their index fingers’ tip. The experimenter then placed the pole and object. Subsequently, the experimenter stepped behind the participant and tapped them on the shoulder as a sign that they were allowed to unblock their ears. Participants then either produced tongue clicks or listened to the loud-speaker clicks (click-train triggered by the experimenter), depending on the condition they were in. Twelve seconds were given for participants to listen to the clicks and echoes and give a response of whether the object was placed in front of them (‘present’) or not (‘absent’). If participants produced their own tongue clicks, the experimenter tapped them on the shoulder again as a sign that time was over for that trial. For the pre-recorded clicks, the end of the click-train signalled that time was over for that trial. If subjects gave no response within those twelve seconds the experimenter requested a judgement. The responses were recorded for each trial. As soon as participants had given a response, they blocked their ears again in order to start with the next trial.

No feedback on the accuracy of response was given. Participants could take breaks as often as they wanted. One session took approximately 90 minutes to complete.

### Data analysis

For sighted participants, we calculated the accuracy of each participant’s responses for each distance (1m vs. 2m), click (self-produced click vs. loud speaker click) and session (1 vs. 2). For the two blind participants we calculated accuracy for each distance and click condition. If participants had answered entirely at random, their accuracy in any condition would have been 0.5.

On the group level, data were analysed using repeated measures ANOVA with ‘session’ (1 vs 2), ‘distance’ (1m vs. 2m) and ‘sound’ (speaker vs. mouth click) as repeated variables. For the two blind people trained in echolocation we analysed their performance on an individual basis in comparison to the group.

To determine if acoustic features of clicks shown in Table 1 were related to performance we ran correlation analyses. For these we correlated individual acoustic features with participants’ performance in mouth-click conditions, and we also ran a multiple-linear regression analysis with individual acoustic features as predictors and participants’ performance in mouth-click conditions as criterion.

## Results

### Group analysis—Sighted Participants

The main effect of ‘session’ was significant (F (1, 26) = 7.899, p = .009) indicating that participants were more accurate in detecting the target object during session 2 (M = .650, SD = 0.119), as compared to session 1 (M = .582; SD = 0.140). Moreover, results showed a significant main effect of sound (F(1, 26) = 8.172, p = .008) indicating that participants detection accuracy was better when they used the loudspeaker (M = .653, SD = 0.161), as compared to when they produced their own tongue clicks (M = .579, SD = 0.093). The analysis also revealed a significant main effect of distance (F(1, 26) = 19.346, p<.001), indicating that subjects’ accuracy in detecting the target object was higher when it was placed at 1m (M = .648, SD = 0.129), as compared to 2m (M = .584, SD = 0.109). In addition, the analysis showed a significant interaction effect between sound and distance (F(1, 26) = 5.549, p = .026) and a significant interaction effect between session, sound and distance (F(1, 26) = 4.398, p = .046). None of the other effects were significant.

**Fig 2:**
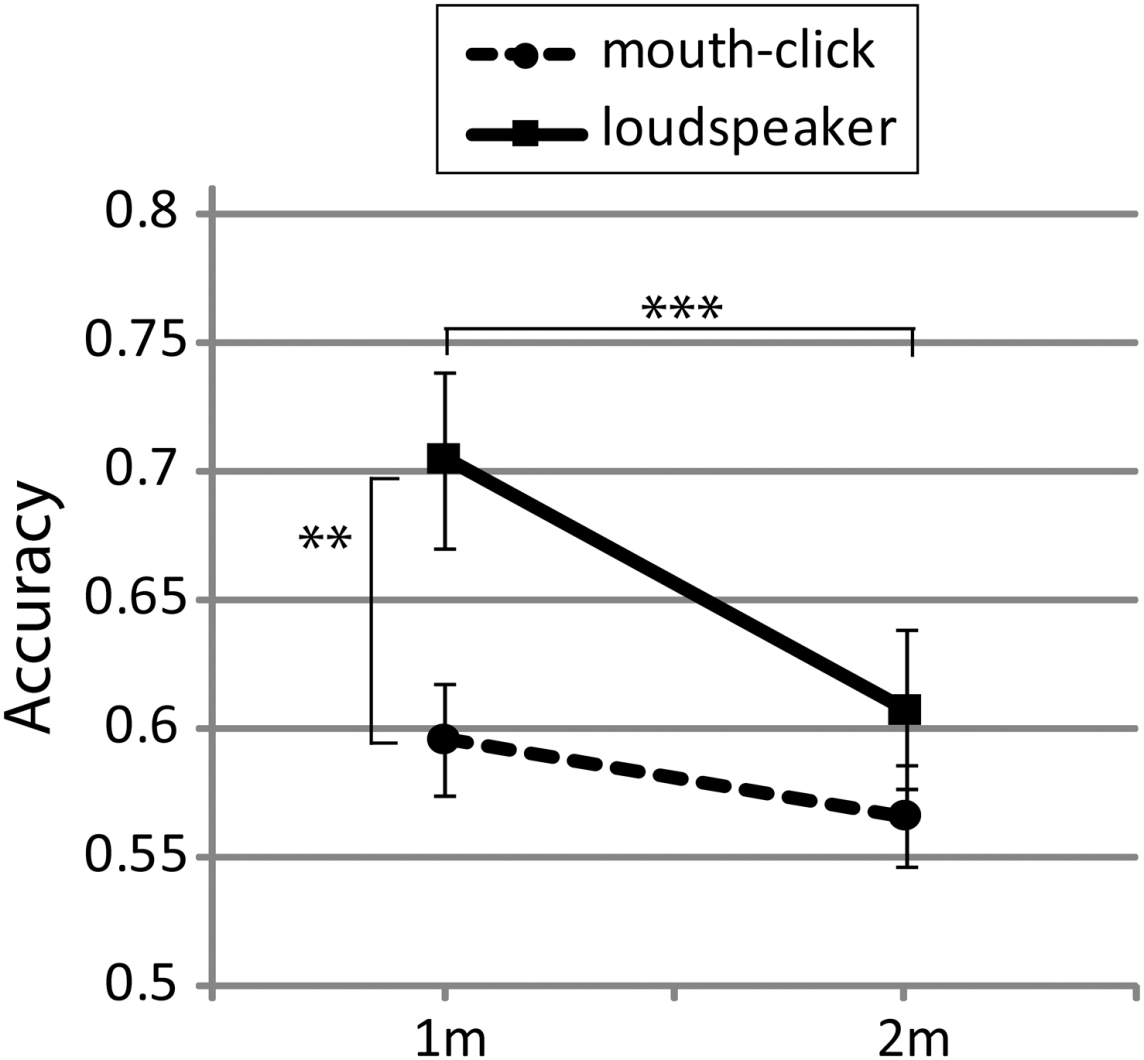
Performance split by distance and sound. Error bars represent SEM across participants. ** p< .01; *** p<.001.

We used paired t-tests (Bonferroni corrected) to follow up the significant interaction effects. The follow up analysis for the sound x distance interaction revealed a significant difference between speaker and mouth-clicks at 1m (t(26) = -3.699; p = .001) but not at 2m (t(26) = -31.303; p<.204). Furthermore, we found that performance was significantly better at 1m as compared to 2m when using the loudspeaker (t(26) = 4.481; p<.001), but not when using mouth clicks (t(26) = 1.51; p = .143). This pattern of results is illustrated in Fig 2.

**Fig 3:**
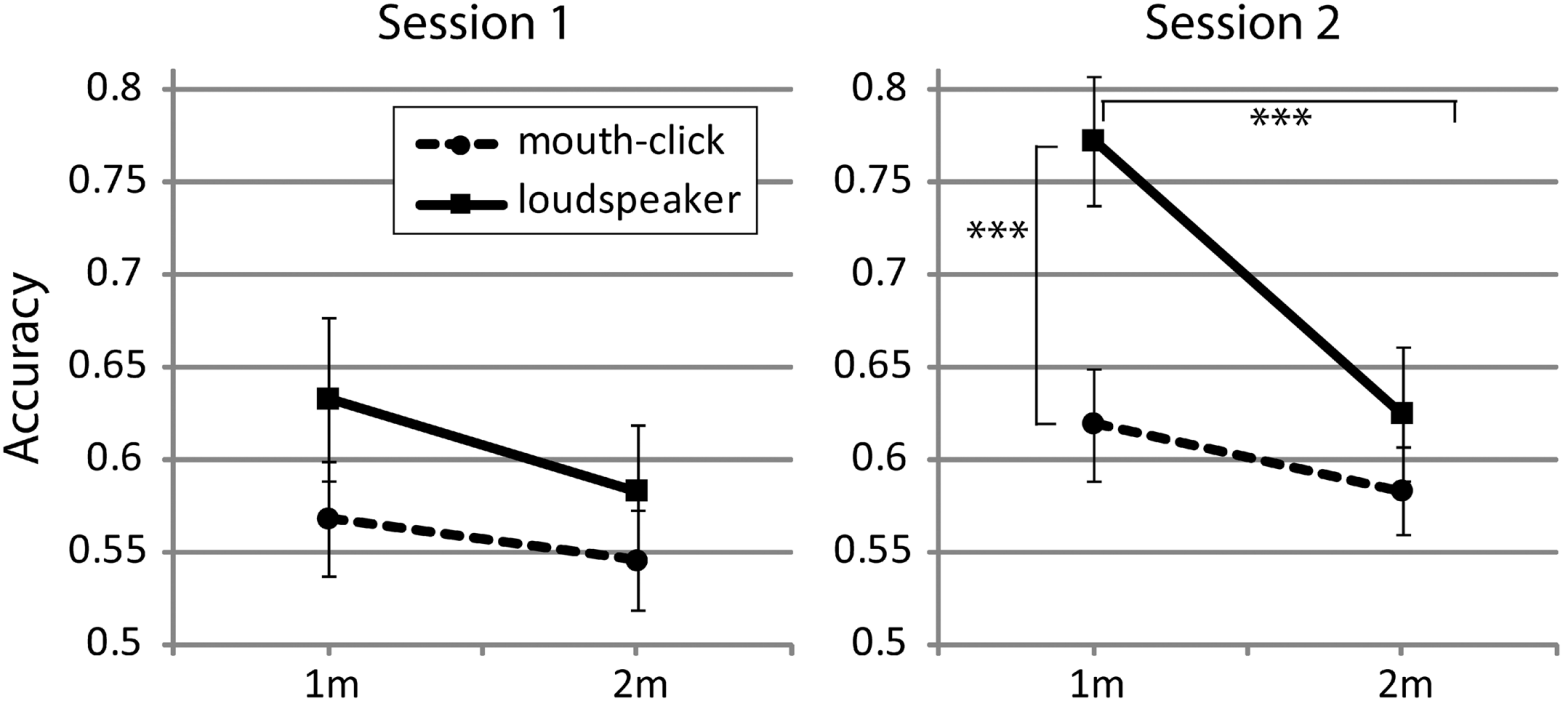
Performance split by session, distance and sound. Error bars represent SEM across participants. *** p<.001.

The follow up analysis for the sound x distance x session interaction confirms these results, but also illustrate that the effects of distance and sound source are only evident in the second session. Specifically, they show that the significant difference between speaker and mouth-click at 1m is only significant for session 2 (t(26) = -4.234;p<.001), but not session 1 (t(26) = -1.542; p = .135), and similarly that better performance at 1m as compared to 2m with the loudspeaker is also only significant for session 2 (t(26) = 5.228;p<.001), but not session 1 (t(26) = 1.925; p = .065). This pattern of results is illustrated in Fig 3.

### Sighted vs. Blind Echolocation Experts

**Fig 4:**
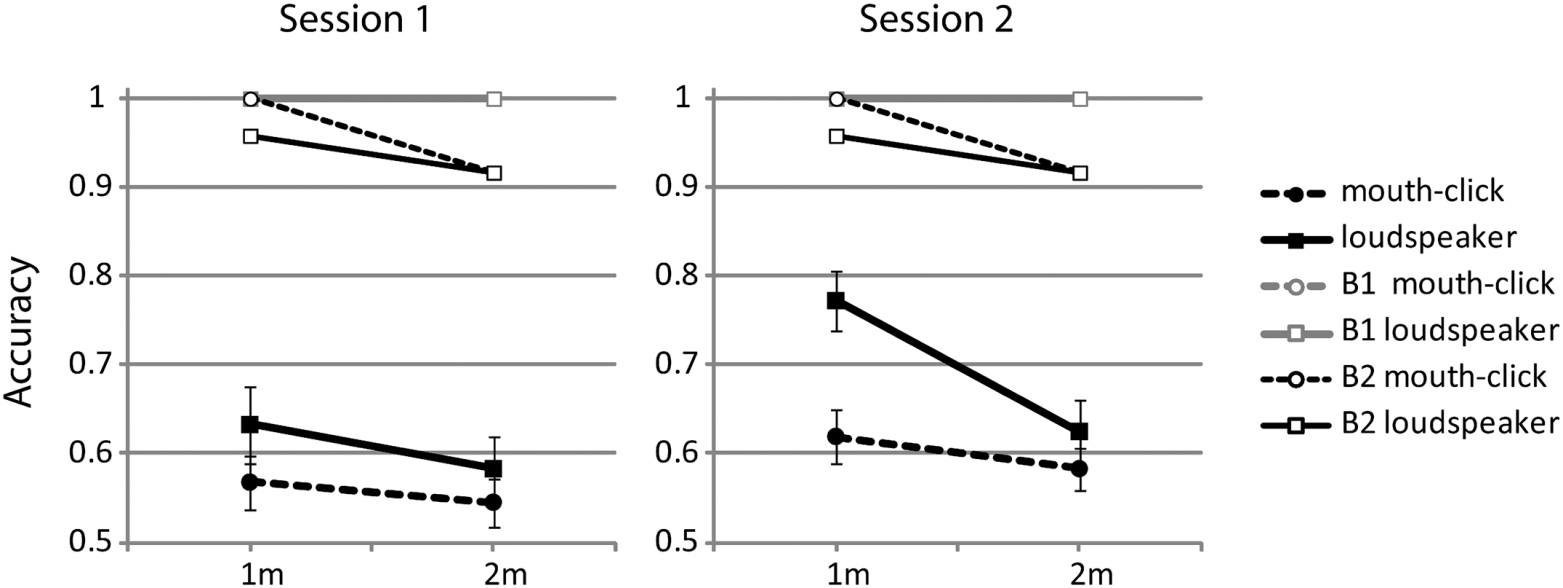
Data for B1 and B2 plotted in comparison to data from sighted participants split by session, distance and sound (i.e. data replotted as from Fig 3). Note that the plot for B1 has two results superimposed. For results of significance tests between sighted participants and B1 and B2 please see Table 2.

Performance of both B1 and B2 plotted together with the data from the group of sighted participants (B1 and B2’s single session performance has been plotted for both session 1 and 2) is shown in Fig 4. It is evident that B1 performs perfectly in all conditions (note that for this reason the plot for B1 has two results superimposed). Thus, B1’s performance is unaffected by distance or sound (mouth click vs. speaker). It is also evident that B2 shows slight variation, but a Chi-square test applied to the distribution of correct responses was non-significant (*χ*^2^(1, N = 91) = .01; p = .919), suggesting that also B2’s performance was the same at 1m and 2m, and for mouth clicks and speaker.

It is also evident that B1 and B2’s performance exceeds performance of sighted participants. To determine if performance differences were significant, we computed modified t-tests which allow comparison of a value of a single case to a group of subjects [21], [22]. Using this procedure, we found that performance of sighted participants was always significantly different from both B1 and B2 when using tongue clicks. In contrast, performance was not significantly different when using a loudspeaker, with the one exception of B1 in session 1 at 2m. The test results are summarized in detail in Table 2.

**Table 2:** Results of modified t-tests comparing performance of B1 and B2 to performance of the sighted sample for each condition.

Condition	Results of modified t-tests
Session 1, 1m, mouth-click	B1: t(26) = 2.65; p = .013*
	B2: t(26) = 2.65; p = .013*
Session 1, 2m, mouth-click	B1: t(26) = 3.216; p = .003**
	B2: 9(26) = 2.626; p = .014*
Session 2, 1m, mouth-click	B1: t(26) = 2.364; p = .026*
	B2: t(26) = 2.364; p = .026*
Session 2, 2m, mouth-click	B1: t(26) = 3.248; p = .003**
	B2: t(26) = 2.599; p = .015*
Session 1, 1m, loudspeaker	B1: t(26) = 1.577; p = .127
	B2: t(26) = 1.397;p = .174
Session 1, 2m, loudspeaker	B1: t(26) = 2.205; p = .037*
	B2: t(26) = 1.764; p = .090
Session 2, 1m, loudspeaker	B1: t(26) = 1.242; p = .225
	B2: t(26) = 1.014; p = .320
Session 2, 2m, loudspeaker	B1: t(26) = 1.952; p = .062
	B2: t(26) = 1.518; p = .141

### Acoustic features of Mouth-Clicks and Performance

**Fig 5:**
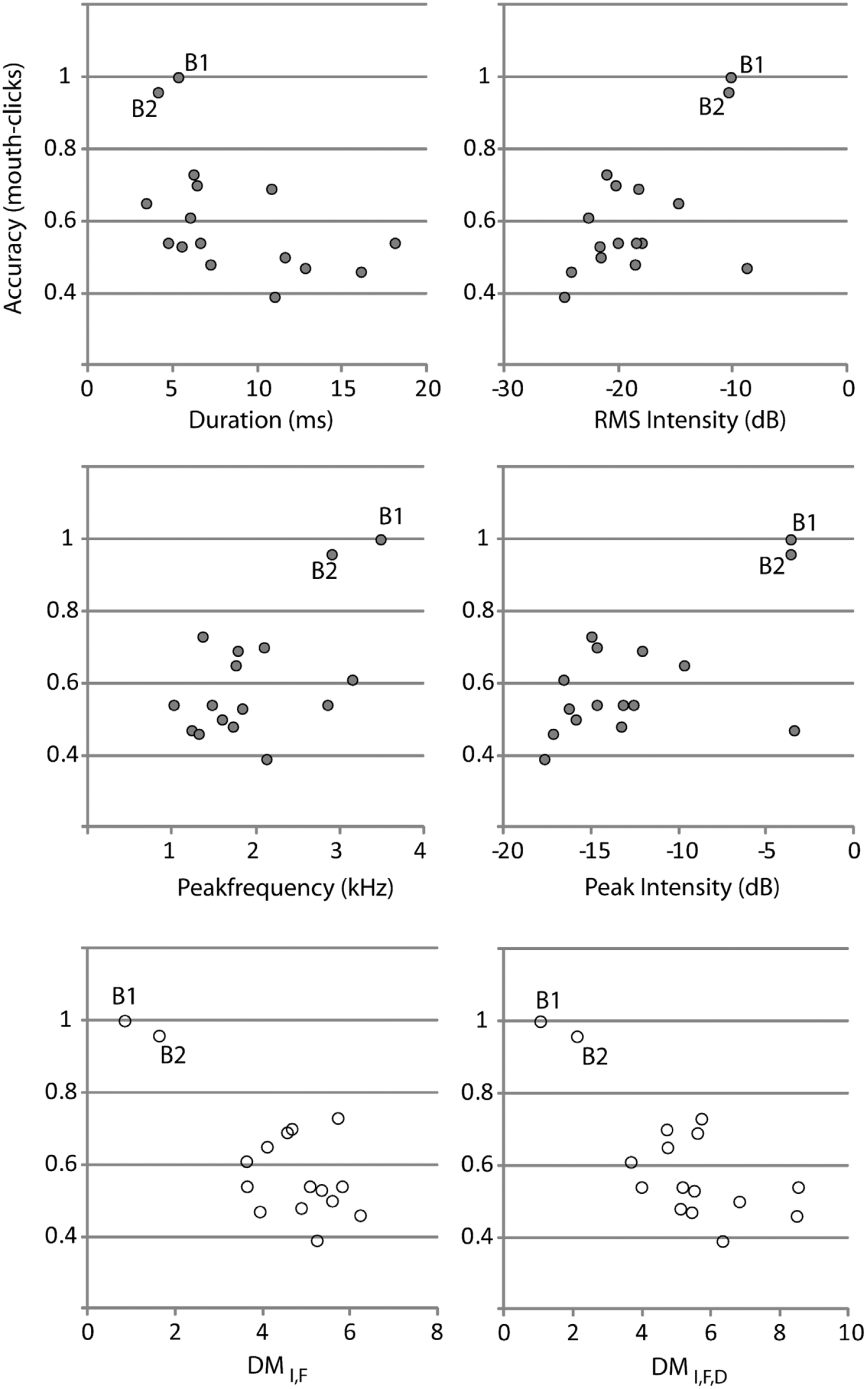
Scatterplots between individual acoustic variables and performance. Data from B1 and B2 are highlighted in the plots.

To investigate the relationship between acoustic features of mouth clicks from a subset (N = 16) of our participants and their performance we adopted a correlation/regression approach. First, we computed individual correlations between each acoustic feature of the clicks and people’s overall accuracy in mouth click conditions (averaged across sessions and distances). Scatterplots are shown in Fig 5. All correlations were significant (duration: r = -.508; p = .045; peak intensity: r = .617; p = .011; RMS intensity: r = .575; p = .02; frequency: r = .589; p = .016). Subsequently, we used stepwise multiple linear regression to determine which variables, or variable combinations, contributed significantly. Using this approach we found that both peak intensity (standardized beta: .499, t(13) = 2.681; p = .019) and peak frequency (standardized beta: .461; t(13) = 2.478; p = .028) had significant positive relationships to overall performance, and that the overall fit was significant (F(2,13) = 8.949; p = .004; R^2^: 0.579). Thus, in our experiment people whose clicks were louder and had higher frequencies performed better when using mouth clicks. When we remove B1 and B2 from the analysis correlations become non-significant (duration: r = -.443; p = .113; peak intensity: r = .067; p = .819; RMS intensity: r = .097; p = .741; frequency: r = .115; p = .695).

To investigate if the similarity of a person’s click to the loud speaker click may be related to how well they did in our experiment, we correlated dissimilarity measures to overall accuracy. We found that the correlation between participants’ overall accuracy and DM_I,F_ was -.768 (p<.001), and for DM_I,F,D_ it was r = -.747 (p = .001). Scatterplots are shown in Fig 5. The data suggest that participants whose clicks were more similar to the loud speaker click did better. As evident from the acoustic statistics shown in Table 1, clicks that were more similar to the speaker also had higher intensity and peak frequencies. When removing B1 and B2 from the analysis correlations become non-significant (DM_I,F_: -.206; p<.480; DM_I,F,D_: r = -.378; p = .183).

## Discussion

Here we tested how well people were able to detect an object in front of them based on acoustic echoes. They could use either mouth clicks or a loudspeaker, and we had both 27 sighted participants new to echolocation and two blind participants with experience in echolocation. We found that sighted participants new to echolocation did generally better when they used a loudspeaker as compared to mouth-clicks, and that this improvement was most pronounced in the second session and at 1m distance. Furthermore, we found that B1 and B2, both of which had experience in echolocation did equally well with mouth clicks and the speaker. Finally, we found that even though B1 and B2 performed generally better than the sighted participants, the difference in performance was only significant when using mouth clicks. In this way, using the speaker enabled sighted participants to approach performance of B1 and B2. Across a subset of 16 of our participants (incl. B1 and B2), those participants whose mouth clicks were more similar to the speaker clicks, and thus had higher peak frequencies and sound intensity, did better.

### Echo-suppression

These results strongly suggest that the use of the loudspeaker did not impair echolocation performance in our experiment. Based on the idea that the active production of a click would lead to reduced echo-suppression [18] we might have expected the opposite pattern of results, namely that participants would have been worse at detecting objects via echoes when they used the speaker, as compared to mouth-clicks. This is expected because if mouth-clicks were to lead to reduced echo suppression, people should do better in echolocation when making mouth-clicks. The fact that we did not observe an advantage of mouth-clicks in our study suggests that reduced echo-suppression during active echolocation as proposed by Wallmeier and colleagues did not drive performance in our experiment.

Nonetheless our task design might have been unsuitable to measure effects of echo-suppression because the sounds that people used in speaker and mouth-click conditions were not identical (compare methods section where we provide data from click measurements). In fact, for the majority of participants whose clicks we measured, we found that their clicks were softer and/or had lower peak frequencies as compared to the clicks made by the speaker.

Thus differences in performance between active clicking and speaker in our study were confounded with differences in the acoustics of the emission itself. In this way then, even though our results suggest that echo-suppression during active echolocation did not drive performance in our experiment, the design of our experiment does not invalidate the hypothesis put forth by [18].

### Acoustic Features

The results of the analyses of acoustic features suggest that (based on individual correlations) intensity, duration and frequency of clicks were related to performance in our experiment. The follow-up multiple linear regression analysis highlighted in particular the contribution of intensity and frequency. Yet, correlations became non-significant when B1 and B2 were excluded from analysis. The latter finding suggests that correlations are driven largely by differences in acoustic click features and performance between sighted participants on the one hand and B1 and B2 on the other.

In our study, perceptual echo-expertise and acoustic features of mouth-clicks are confounded because B1 and B2 not only have clicks that are typically shorter, higher, and more intense compared to those of sighted participants, but they also have more experience in perceiving and processing echoes. Thus, we cannot be sure if the correlations we observe are indicative of an association between performance and acoustic features of clicks or if they are indicative of an association between performance and perceptual-cognitive echo-expertise. Nonetheless, there is previous research that is generally consistent with what we found in regards to frequency and intensity. For example, [23] and [24] found that people’s perception of lateral position was better with high-pass (>2kHz) as compared to low pass (<2Khz) stimuli. They also found that performance improved with increasing sound level. Nonetheless, the stimuli they used were noise stimuli, not clicks. Interestingly, with respect to emission duration it has been reported that people tend to do better with longer sounds. For example, [23] found that performance to localize the lateral position of an object increased as stimulus duration increased from 10–400ms. Similarly, [25] found that people’s ability to determine the presence of an object increased as stimulus duration increased from 5ms to 50ms to 500 ms. In our experiment shorter clicks were associated with better performance, however, which may seem at odds with these previous findings. This can potentially be explained considering that the magnitude of duration differences that we observed across participants were far below those duration differences used by [23] or [25]. Furthermore, we did not use noise stimuli, but clicks. In sum, future work should investigate the issue of acoustic click features more systematically, and our results as well as the other work discussed above suggest that duration, frequency and intensity should be features to consider in this context.

### Generalization to other Tasks

The task we used here was a simple object detection task. Future work is needed to determine how the results generalize to more complex scenarios and tasks.

### Assistive Technology

The main goal of our work was to test if people could successfully echolocate using a loudspeaker, and how it would compare to when they used their own mouth- clicks. We addressed this question because of its high relevance to developers of assistive devices, which work based on technology rather than people making their own emissions. Here we found that the use of a loudspeaker enabled people who had no experience in echolocation to improve their performance as compared to when they used their own mouth clicks, and that this advantage was most pronounced at 1m distance and in the second testing session. Most importantly, we also found that these ‘echo naïve’ people, when using the loudspeaker, were able to perform similar (i.e. not significantly different) to two echolocation experts, i.e. people who have longstanding expertise in echolocation. Finally, for these two echolocation experts the use of a loudspeaker did not make any difference, i.e. they performed equally well in all conditions. This suggests that the use of technology as simple as a head-worn loudspeaker making audible clicks enables people to perform better or just as well as when using mouth-clicks.

As mentioned in the introduction, various technological assistive devices for people with vision impairments have been developed based on the echolocation principle [6]–[15]. The devices range in their complexity and purpose, but all have in common that they generate the emission and feed a more or less processed signal back to the user. The advantage of technological assistive devices is that they can, for example, achieve greater spatial resolution by working in the ultrasonic range, but our current results suggest that even a tool as simple as a head worn acoustic loudspeaker may facilitate echolocation. Natural echolocation offers advantages in terms of ease of access, sturdiness, and low cost. Future research will determine the degree to which assistive technology may or may not supersede natural echolocation.

## Conclusion

Our study is the first to directly compare people’s performance in an echolocation task when they used their mouth or a head-worn loudspeaker to make clicks. Performance was either the same or better with the loudspeaker. This result is encouraging for the development of assistive technology based on echolocation.
